# Monoblock and Modular Dual-Mobility Constructs Versus Standard Cups with Femoral Heads ≥ 36 mm in Cementless Total Hip Arthroplasty for Femoral Neck Fracture: A 10-Year Comparative Registry Study of 10-Year Survival Estimates

**DOI:** 10.3390/medicina62081592

**Published:** 2026-08-19

**Authors:** Serena Santoro, Barbara Bordini, Monica Cosentino, Stefano Lucchini, Rosalinda Ventimiglia, Danilo Donati, Francesco Castagnini, Francesco Traina

**Affiliations:** 1 Ortopedia-Traumatologia e Chirurgia Protesica e dei Reimpianti D’Anca e di Ginocchio, IRCCS Istituto Ortopedico Rizzoli, 40136 Bologna, Italy; serena.santoro@ior.it (S.S.); stefano.lucchini@ior.it (S.L.); rosalinda.ventimiglia@ior.it (R.V.); francesco.traina@ior.it (F.T.); 2 Laboratorio di Tecnologia Medica, IRCCS Istituto Ortopedico Rizzoli, 40136 Bologna, Italy; barbara.bordini@ior.it; 3 Direzione Scientifica, IRCCS Istituto Ortopedico Rizzoli, 40136 Bologna, Italy; monica.cosentino@ior.it; 4 Riabilitazione della Mano ed Arto Superiore, Azienda Ospedaliero Universitaria Policlinico Modena, 40126 Modena, Italy; danilo.donati@unimore.it; 5 Clinical and Experimental Medicine PhD Program, University of Modena and Reggio Emilia, 41121 Modena, Italy; 6 Dipartimento di Scienze Biomediche e Neuromotorie Dibinem, Università di Bologna, 40126 Bologna, Italy

**Keywords:** dual mobility, dislocation, total hip arthroplasty

## Abstract

*Background and Objectives:* Dual-mobility cups are commonly used to reduce instability after total hip arthroplasty (THA) for femoral neck fracture (FNF), but their advantage over contemporary large-head standard cups remains uncertain. This registry study compared revision-related survival estimates up to 10 years among monoblock dual-mobility cups, modular dual-mobility constructs, and standard cups with femoral heads ≥ 36 mm in cementless THA for FNF. *Materials and Methods:* We retrospectively analyzed regional residents undergoing cementless, non-metal-on-metal primary THA for FNF. The cohort included 4827 THAs: 1220 monoblock dual-mobility cups, 408 modular dual-mobility constructs, and 3199 standard cups with ≥36 mm heads. Groups differed in age and sex distribution, with modular dual mobility used in older patients and both dual-mobility constructs used more often in women. Kaplan–Meier analysis estimated survival up to 10 years using aseptic failure and revision for dislocation/instability as endpoints. Cox models estimated adjusted hazard ratios, and cumulative incidence of aseptic failure was assessed with death as a competing event. *Results:* Kaplan–Meier-estimated 10-year aseptic failure-free survival was 94.4% for monoblock dual mobility, 96.8% for modular dual mobility, and 95.4% for standard cups (*p* = 0.815). Cup construct was not independently associated with aseptic failure: compared with standard cups, the HR was 1.12 for monoblock dual mobility (95% CI, 0.75–1.67) and 1.34 for modular dual mobility (95% CI, 0.71–2.52). Kaplan–Meier-estimated 10-year survival free from revision for dislocation/instability was 99.6%, 99.2%, and 99.3%, respectively (*p* = 0.620), with no independent effect of cup construct. Male sex increased the risk of both aseptic failure and revision for dislocation/instability. Periprosthetic fracture was the leading cause of aseptic revision in both dual-mobility groups, whereas failures were more evenly distributed in the standard cup group. Death-adjusted cumulative incidence of aseptic failure was similar across constructs (*p* = 0.919). *Conclusions:* Neither monoblock nor modular dual mobility improved revision-related outcomes compared with standard cups using femoral heads ≥ 36 mm. Late survival estimates, particularly for modular dual mobility, should be interpreted cautiously because of the limited number of patients remaining at risk.

## 1. Introduction

Dislocation remains a major reason for revision after total hip arthroplasty (THA), accounting for approximately 17% of THA failures in the United Kingdom [1,2]. This problem is amplified after THA for femoral neck fracture (FNF), where instability rates may be up to fivefold higher than after elective THA for osteoarthritis [1,2]. Developed by Bousquet in the 1970s aiming to reduce THA instability, dual-mobility cups use a mobile polyethylene liner articulating with both the femoral head and the acetabular shell, thereby increasing the effective head–neck ratio, range of motion before impingement, and jump distance [3,4,5]. Experimental data reported gains of approximately 30° in flexion, 15° in adduction, and 22° in external rotation compared with conventional bearings, although recent modeling challenged the assumption that dual mobility provides a uniformly larger impingement-free range of motion across all functional positions [1,4]. These theoretical advantages led to increasing use in higher-risk patients: in the Dutch Arthroplasty Register, primary dual-mobility THA increased from 0.8% in 2010 to 2.6% in 2016, with greater use in patients with FNF and higher ASA status compared with unipolar cups [6]. Consistently, since 2007, several dual-mobility systems have been FDA-cleared through the 510(k) substantial-equivalence pathway for THA indications explicitly including dislocation risk and FNF [7,8].

Clinical evidence in FNF, however, remains less definitive than the biomechanical and regulatory rationale would suggest. A 2025 meta-analysis of 13 studies and 21,585 patients reported lower risks of dislocation (RR, 0.47; 95% CI, 0.34–0.65) and revision (RR, 0.77; 95% CI, 0.67–0.89) with dual-mobility THA, but pooled randomized and observational studies, different fixation methods, surgical approaches, implant designs, and conventional head sizes limit interpretation [9]. Registry data are even more cautious. In the Australian Registry, Hoskins et al. analyzed 16,692 THAs for FNF, including 8582 standard-head, 5820 large-head, 1778 dual-mobility, and 512 constrained constructs, and found no adjusted difference in all-cause revision at 7 years. Large heads reduced revision for dislocation compared with standard heads (HR, 0.60; 95% CI, 0.40–0.80), whereas dual mobility reduced revision for dislocation only during the first three postoperative months (HR, 0.30; 95% CI, 0.10–0.70), without a sustained survival advantage [10]. Similarly, in a propensity-score-matched Swedish registry study, Rogmark and Nauclér compared 2242 dual-mobility THAs with 6726 conventional THAs and found similar 5-year revision rates after posterior approach (4.7% versus 4.8%) and lateral approach (2.3% versus 3.7%); revision for dislocation was also low and comparable [11]. In neurologically impaired fracture patients, Cnudde et al. reported lower 1-year dislocation after dual-mobility THA than after conventional THA with heads < 32 mm (2.7% versus 8.8%), but with no difference in reoperation or revision-free survival [12].

Thus, the comparative value of dual mobility THA in FNF remains uncertain, particularly against contemporary standard cups with large heads. Moreover, most studies do not distinguish monoblock from modular dual-mobility cups, despite differences in shell design, liner fixation, screw-fixation options, and modular junctions [13,14]. Therefore, a regional arthroplasty registry study was designed, aimed to compare three acetabular bearing constructs in cementless primary THA for FNF: monoblock dual-mobility cups, modular dual-mobility constructs, and standard cups with femoral heads ≥ 36 mm. The aims were to compare Kaplan–Meier-estimated survival rates up to 10 years, using aseptic failures and dislocation/instability as endpoints, among the three constructs, to estimate adjusted hazard ratios for aseptic failure and revision for dislocation/instability, to assess the cumulative incidence of aseptic failure with death as a competing event, and to describe reasons for revision. We hypothesized that neither monoblock nor modular dual mobility would provide superior survival rates up to 10 years compared with THA using femoral heads ≥ 36 mm.

## 2. Materials and Methods

Institutional review board approval was not required for this study due to the standard practice of data anonymization and registry data collection.

The RIPO (Registro dell’Implantologia Protesica Ortopedica) registry was used to identify cementless primary THA performed for FNF [15,16]. The registry has been collecting data on primary and revision arthroplasty procedures since January 2000 and records patient demographics, diagnosis, implant characteristics (batch and codes), fixation, bearing surfaces, surgical approach, and reasons for revision. The RIPO registry covers approximately 4.5 million residents and includes 69 orthopedic facilities in the Emilia–Romagna region [15,16,17]. For each primary and revision arthroplasty, surgeons are required to complete standardized forms recording patient demographics, implant characteristics, and surgical details [15,16,17,18]. Registry data are routinely cross-checked against additional administrative and clinical databases, resulting in an estimated capture rate of 98%, comparable with that of major national arthroplasty registries [18]. The remaining 2% of missing data is mainly attributable to incomplete reporting or non-adherence to registry submission procedures [18]. RIPO is also a member of the International Society of Arthroplasty Registries (ISAR) [19].

A registry study on THA performed for FNF was conducted, collecting data from 1 January 2000 to 31 December 2022. As 82% of the THA after FNF was cementless, in order to reduce confounding factors, only cementless implants were included.

So, the inclusion criteria were as follows: cementless primary THA, diagnosis of FNF, resident status within the Emilia–Romagna region, and availability of complete implant data allowing reliable classification of the acetabular construct.

The exclusion criteria were as follows: revision total hip arthroplasty, hemiarthroplasty, resurfacing hip arthroplasty, cemented or hybrid fixation, constrained liners, metal-on-metal bearings, fixed-bearing constructs with femoral heads < 36 mm, and implants with incomplete or ambiguous data.

**Figure 1:**
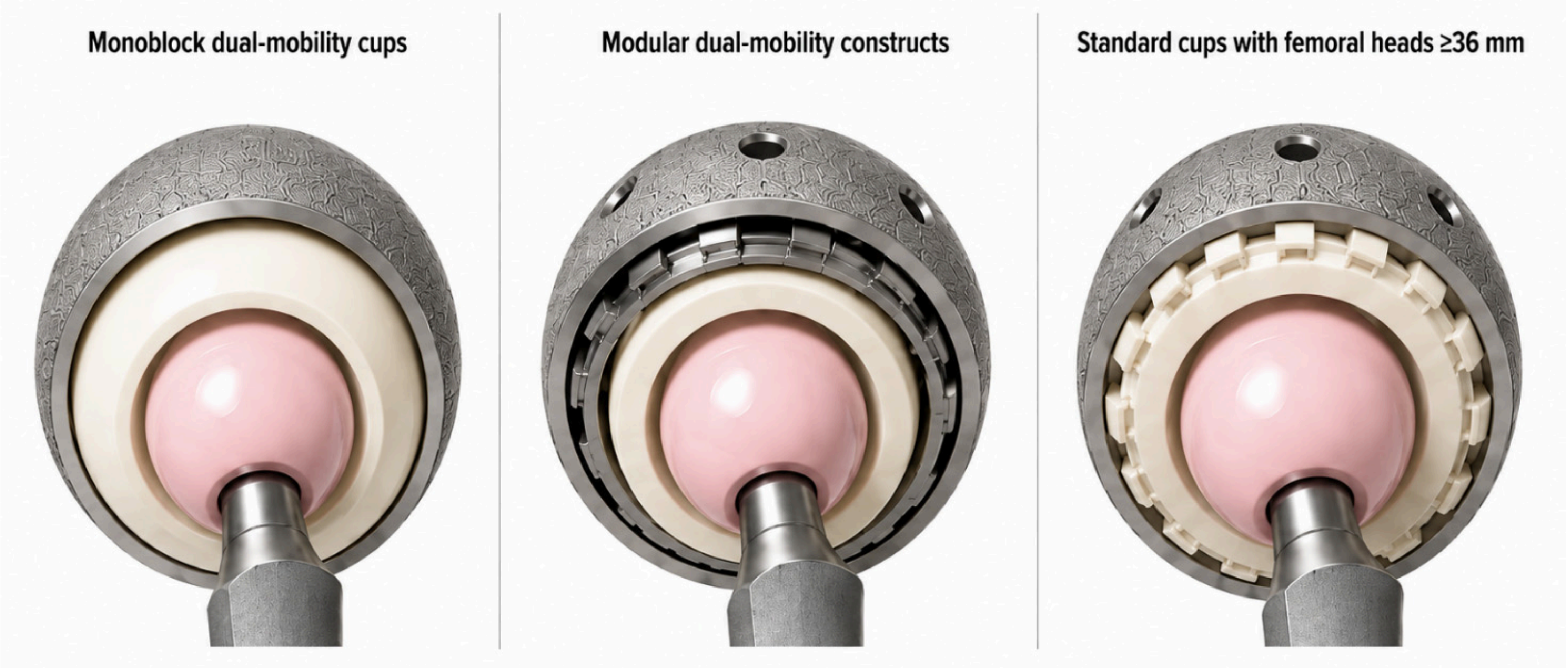
Illustrative examples of the three acetabular construct groups analyzed in the study: monoblock dual-mobility cups, modular dual-mobility constructs, and standard cups with femoral heads ≥ 36 mm.

After application of the inclusion and exclusion criteria, 4827 cementless primary THA were included. According to manufacturer-defined implant characteristics and bearing configuration, three mutually exclusive acetabular construct groups were identified (Figure 1).

The first group included monoblock dual-mobility cups, defined as acetabular components designed only for dual-mobility articulation, with a mobile polyethylene liner interposed between the femoral head and the monoblock acetabular cup with internal finishing for articular coupling [3,4,5,13].

The second group included modular dual-mobility constructs, defined as standard acetabular shells designed to accept different fixed liners but, in the cases analyzed, combined with a modular dual-mobility metal insert to obtain a dual-mobility articulation [13,14].

The third group included standard cups with femoral heads ≥ 36 mm, defined as standard acetabular shells with a fixed liner and a large femoral head ≥ 36 mm [10].

Of the 4827 cementless primary total hip arthroplasties included, 1220 THAs were classified as monoblock dual-mobility cups, 408 THAs as modular dual-mobility constructs, and 3199 THAs as standard cups with femoral heads ≥ 36 mm.

Demographic, implant-related, and surgical variables were extracted from the registry: age at surgery, age class, sex, body mass index, femoral head material, femoral neck type, and surgical approach.

According to the study aims, Kaplan–Meier analysis was used to estimate survival up to 10 years with aseptic failure and revision for dislocation/instability as separate endpoints. Cox proportional hazard models were used to estimate adjusted hazard ratios for both endpoints, while cumulative incidence analysis with death as a competing event was performed for aseptic failure. Reasons for revision were summarized descriptively as frequencies and percentages within each construct group.

### Statistical Analysis

The statistical analysis was done using R Core Team (4.3, 2023) [20]. Continuous variables were reported as median, interquartile range, minimum and maximum, and mean with standard deviation. Categorical variables were reported as absolute numbers and percentages. Baseline demographic, surgical, and implant-related features were compared among the three groups. Data distribution was assessed using the Shapiro–Wilk test. Continuous variables were compared using Welch’s one-way analysis of variance. Categorical variables were compared using Pearson’s chi-square test, while continuous variables were compared using one-way analysis of means. Implant survival was calculated and plotted according to the Kaplan–Meier method, with revision for aseptic failure (every revision performed for other reasons than periprosthetic infection) and for dislocation/instability within 10 years as the endpoint. The implant was considered “surviving” until the exchange of any component. Confidence intervals (CIs) of 95% were calculated. For unrevised implants, survival times were recorded as of the last observation date (date of death or 31 December 2022). Survival curves were compared using the log-rank test. Cox proportional hazard regression models were used to estimate hazard ratios and 95% confidence intervals for aseptic failure and dislocation/instability. The standard cup with femoral heads ≥ 36 mm group was used as the reference category. The model included age, sex, and acetabular construct group. The single/dual taper neck and surgical approach were added when dislocation/instability was adopted as the endpoint. A competing-risk analysis was performed considering death as a competing event. Cumulative incidence curves were calculated and compared using Gray’s test. A *p*-value < 0.05 was considered statistically significant.

ChatGPT by OpenAI (version 5.5) was used for language editing, rephrasing, and improving manuscript readability, as well as for assistance with non-data-based illustrative figures. It was not used for data analysis, interpretation, or generation of scientific conclusions. All content was reviewed and approved by the authors.

## 3. Results

Baseline characteristics of the three cohorts are reported in Table 1. All these variables but body mass index showed different distribution among the cohorts.

The three most frequently used cup models were Dualis (Bioimpianti, Peschiera Borromeo, Italy; 211/1220, 17.3%), Easy (Lima Corporate, San Daniele, Italy; 149/1220, 12.2%), and Quattro HAP PnP (Groupe Lépine, Genay, France; 140/1220, 11.5%) in the monoblock dual-mobility group; Trident PSL HA (Howmedica Osteonics/Stryker, Mahwah, NJ, US; 213/408, 52.2%), Fixa Ti-Por (Adler Ortho, Cormano, Italy; 60/408, 14.7%), and Jump System Traser (Permedica, Merate, Italy; 50/408, 12.3%) in the modular dual-mobility group; and Fixa Ti-Por (Adler Ortho, Cormano, Italy; 915/3199, 28.6%), G7 PPS (Zimmer Biomet, Warsaw, IN, US; 306/3199, 9.6%), and R3 (Smith & Nephew, Memphis, TN, US; 266/3199, 8.3%) among standard cups with ≥36 mm heads. After censoring follow-up at 10 years, median follow-up was 3.2 years (IQR, 0.8–6.1) in the monoblock dual-mobility group, 2.1 years (IQR, 0.8–3.8) in the modular dual-mobility group, and 4.5 years (IQR, 1.9–8.2) in the standard cup group. Mean follow-up was 3.9 ± 3.3 years, 2.6 ± 2.2 years, and 4.9 ± 3.4 years, respectively.

### 3.1. Survival Free from Aseptic Failure

**Figure 2:**
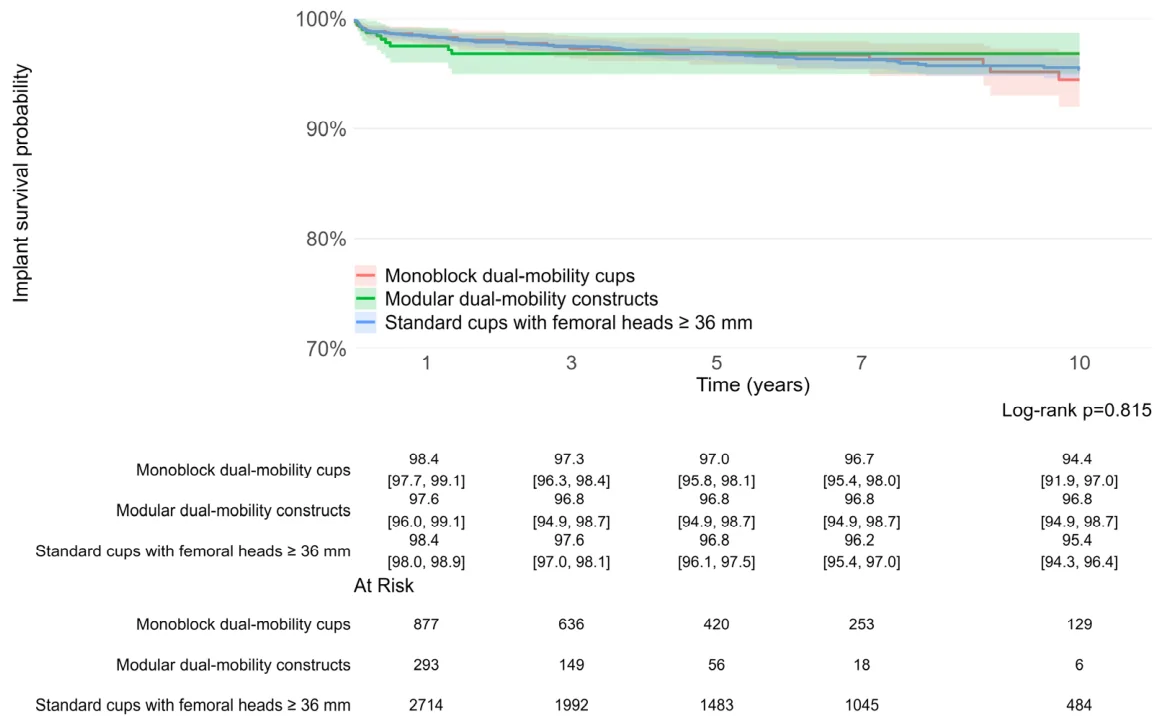
Kaplan–Meier survival curves of the three cohorts were comparable up to 10 years.

Kaplan–Meier analysis showed no statistically significant difference in survival rates among the three groups (aseptic failure as endpoint, log-rank *p* = 0.815) (Figure 2). Up to 10 years, implant survival was 94.4% (95% CI, 91.9–97.0%) in the monoblock dual-mobility group, 96.8% (95% CI, 94.9–98.7%) in the modular dual-mobility construct group, and 95.4% (95% CI, 94.3–96.4%) in the standard cup group.

### 3.2. Reasons for Aseptic Failure

The distribution of reasons for aseptic failure is reported in Table 2. Overall, 142 aseptic failures occurred within 10 years: 33 events in the monoblock dual-mobility group, 11 events in the modular dual-mobility group, and 98 events in the standard cup group. Periprosthetic fracture was the most frequent cause of aseptic failure in the monoblock and modular dual-mobility group (33.3% and 54.5%, respectively). Cup aseptic loosening was the second most frequent in the monoblock cohort (24.2%), whereas dislocation was the second most frequent in the modular group (27.3%). In the standard cup group, the most frequent causes were periprosthetic fracture (25.5%), dislocation/primary instability (21.4%), and stem aseptic loosening (20.4%).

### 3.3. Survival Free from Revision for Dislocation/Instability

**Figure 3:**
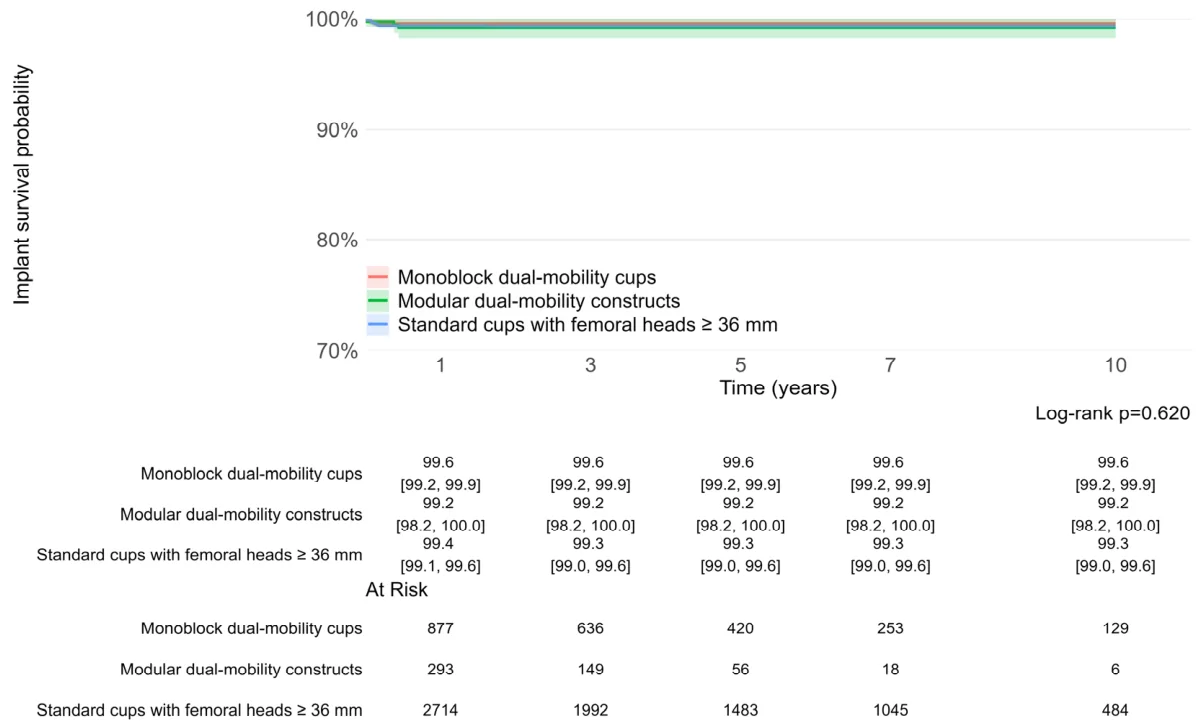
Kaplan–Meier survival curves for revision due to dislocation/instability within 10 years according to acetabular construct.

Kaplan–Meier analysis using revision for dislocation/instability as the endpoint showed very high and comparable estimated 10-year survival across groups (log-rank *p* = 0.620) (Figure 3). Estimated 10-year revision-free survival was 99.6% (95% CI, 99.2–99.9%) for monoblock dual mobility, 99.2% (95% CI, 98.2–100%) for modular dual mobility, and 99.3% (95% CI, 99.0–99.6%) for standard cups.

### 3.4. Cox Regression Analysis for Aseptic Failure

Cox regression analysis was performed with the endpoint as aseptic failure. Age was not associated with aseptic failure (HR 1.00, 95% CI 0.98–1.01; *p* = 0.721). Male sex was associated with a higher risk of aseptic failure compared with female sex (HR 1.60, 95% CI 1.14–2.25; *p* = 0.007). Using standard cups with femoral heads ≥ 36 mm as the reference group, no statistically significant difference in the risk of aseptic failure was found for monoblock dual-mobility cups (HR 1.12, 95% CI 0.75–1.67) or modular dual-mobility constructs (HR 1.34, 95% CI 0.71–2.52) (*p* = 0.632).

### 3.5. Cox Regression Analysis for Revision for Dislocation/Instability

In the multivariable Cox model with endpoint revision due to dislocation/instability within 10 years, male sex was associated with increased risk (HR, 2.83; 95% CI, 1.27–6.30; *p* = 0.008), whereas age was not significant (HR, 1.03; 95% CI, 0.99–1.07; *p* = 0.157). Surgical approach was not associated with revision for dislocation/instability (global *p* = 0.890): compared with anterior/other approaches, the lateral approach had an HR of 1.27 (95% CI, 0.36–4.47) and posterolateral approach an HR of 1.37 (95% CI, 0.37–5.01). Neck modularity was also not significant (HR, 1.54; 95% CI, 0.64–3.69; *p* = 0.349). Finally, cup construct was not independently associated with revision for dislocation/instability (global *p* = 0.724): compared with monoblock dual mobility, modular dual mobility had an HR of 1.68 (95% CI, 0.40–7.07), and standard cups with heads ≥ 36 mm had an HR of 1.42 (95% CI, 0.49–4.15).

### 3.6. Competing-Risk Analysis

**Figure 4:**
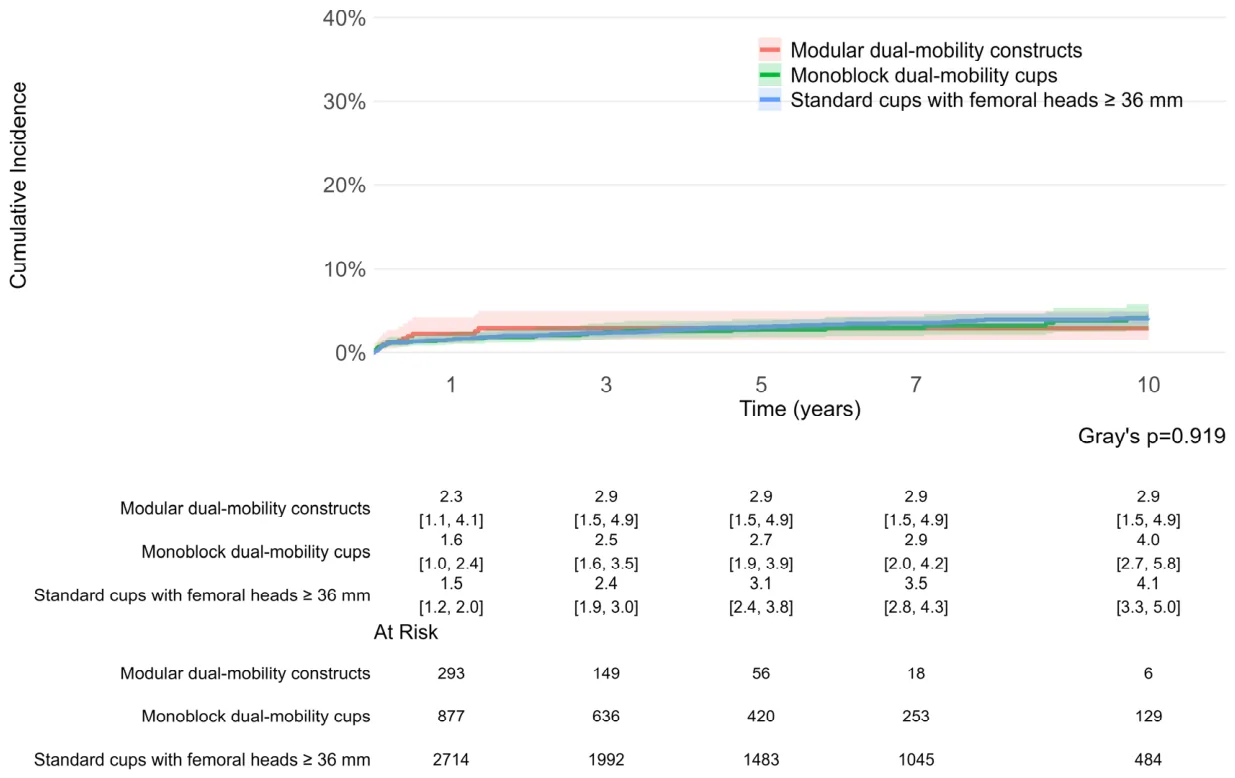
Cumulative incidence curves for aseptic failure within 10 years according to acetabular construct, considering death as a competing event.

When death was considered as a competing event, the cumulative incidence of aseptic failure did not differ significantly among the three groups (Gray test *p* = 0.919) (Figure 4). Up to 10 years, the cumulative incidence was 4.0% in the monoblock dual-mobility group, 2.9% in the modular dual-mobility group, and 4.1% in the standard cup group.

## 4. Discussion

This registry study found no evidence that monoblock or modular dual-mobility cups provided superior aseptic survivorship compared with standard cups using femoral heads ≥ 36 mm in cementless THA for FNF up to 10 years. Survival free from revision for dislocation/instability was also high and comparable across constructs. In adjusted Cox analyses, cup construct was not independently associated with either aseptic failure or revision for dislocation/instability, whereas male sex was associated with increased risk for both endpoints. Reasons for aseptic revision differed descriptively across groups, but dislocation represented only a minority of failures and did not drive overall survivorship. Death-adjusted competing-risk analysis confirmed similarly low cumulative incidence of aseptic failure across constructs, supporting the main hypothesis that neither monoblock nor modular dual mobility improves long-term revision-related outcomes over standard cups with ≥36 mm heads in this setting.

The first aim was to compare survival rates with aseptic failure as the endpoint up to 10 years. Survivorship was 94.4% for monoblock dual mobility, 96.8% for modular dual mobility, and 95.4% for standard cups with ≥36 mm heads, with no between-group difference (*p* = 0.815). These findings are consistent with the Australian Registry study by Hoskins et al., which analyzed 16,692 THA for FNF (8582 standard-head, 5820 large-head, 1778 dual-mobility, and 512 constrained constructs): no adjusted difference in all-cause revision was found at 7 years [10]. Our results also align with Rogmark and Nauclér, who compared 2242 dual-mobility THAs with 6726 conventional THA in a Swedish propensity-score-matched registry cohort and found similar 5-year revision rates after the posterior approach (4.7% versus 4.8%) and lateral approach (2.3% versus 3.7%) [11]. The analysis of revision for dislocation/instability as the endpoint reinforces the same interpretation. Although dual mobility has been associated with lower dislocation risk in large FNF populations, including the meta-analysis by Santiago et al. (RR, 0.47; 95% CI, 0.34–0.65) and the neurologically impaired cohort by Cnudde et al. (2.7% with dual mobility versus 8.8% with conventional THA using heads < 32 mm), this advantage was not observed in our study when the comparator was a large-head construct [9,12]. This suggests that the anti-instability benefit of dual mobility may be not so relevant when compared with modern large-head standard cups, at least in non-specifically selected populations.

The adjusted hazard analysis confirmed the previous findings. Compared with standard cups using ≥36 mm heads, monoblock dual mobility had an HR of 1.12 (95% CI, 0.75–1.67) and modular dual mobility an HR of 1.34 (95% CI, 0.71–2.52), with no independent effect of construct type. Hoskins et al., adjusting for age, sex, femoral fixation, and head size where appropriate, similarly found no all-cause revision advantage for dual mobility over large heads (HR, 0.91; 95% CI, 0.69–1.21; *p* = 0.511), nor for dual mobility versus standard heads (HR, 0.90; 95% CI, 0.68–1.19; *p* = 0.459) [10]. In the multivariable model for revision due to dislocation/instability, cup construct, surgical approach, and stem-neck modularity were not independently associated with the endpoint, whereas male sex was the only significant risk factor. This finding completes the data by Hoskins et al., showing that dual mobility reduced revision for dislocation only during the first 3 months compared with standard heads (HR, 0.30; 95% CI, 0.10–0.70), while large heads showed a more sustained protective effect versus standard heads (HR, 0.60; 95% CI, 0.40–0.80) [10]. Rogmark and Nauclér similarly found low and comparable 5-year revision for dislocation after dual mobility and conventional THA, both after the posterior approach (1.3% versus 2.2%) and lateral approach (0.4% versus 0.7%) [11]. These registry findings differ from the broader meta-analytic signal reported by Santiago et al., where dual mobility reduced overall dislocation risk (RR, 0.47; 95% CI, 0.34–0.65), and from Cnudde et al., where dual mobility showed lower 1-year dislocation than conventional THA with heads < 32 mm in neurologically impaired patients (2.7% versus 8.8%) [9,12]. Taken together, these data suggest that dual mobility may be most protective against dislocations when compared with small-head conventional THA or in selected high-risk patients, whereas its independent advantage becomes less evident when the comparator is a large-head standard cup.

The pattern of aseptic failure was also informative. Up to 10 years, periprosthetic fracture was the leading reason for aseptic revision in both monoblock and modular dual-mobility groups, accounting for 33.3% and 54.5% of aseptic failures, respectively. Dislocation represented 27.3% of revisions in the modular group and was less dominant in the monoblock group. In the standard cup group, failures were more evenly distributed, with periprosthetic fracture (25.5%), dislocation (20.4%), and stem aseptic loosening (20.4%) contributing similarly. This differs from Hoskins et al., where dislocation was the leading cause of all-cause revision overall and accounted for 40% of revisions with standard heads, 23% with large heads, and 27% with dual mobility, while infection accounted for 15%, 21%, and 29%, respectively [10]. The difference likely reflects endpoint definition and follow-up: our aseptic endpoint up to 10 years captures later mechanical events, whereas shorter follow-up and all-cause revision analyses are more influenced by early instability and infection. Although dual-mobility constructs and modular-neck stems introduce additional interfaces that may generate wear debris, corrosion products, or mechanical complications, the present study detected no significant adverse effect attributable to construct type or neck modularity [21,22].

Accounting for death as a competing event is very useful in THA after FNF, because mortality may preclude later revision and Kaplan–Meier estimates may overstate absolute implant-failure risk. In our cohort, estimated 10-year cumulative incidence of aseptic failure was low and similar: 4.0% for monoblock dual mobility, 2.9% for modular dual mobility, and 4.1% for standard cups with ≥36 mm heads (Gray *p* = 0.919). Hoskins et al. acknowledged death as a competing risk but censored it in Kaplan–Meier analysis [10]. Rogmark and Nauclér performed a competing-risk sensitivity analysis at 5 years, with results close to Kaplan–Meier estimates but did not assess 10-year aseptic failure across monoblock dual mobility, modular dual mobility, and large-head standard constructs [11]. Our findings therefore suggest that the absence of a dual-mobility advantage was not driven by mortality censoring.

This study has limitations. Residual confounding by indication cannot be excluded, because dual-mobility cups may have been selected for patients considered at higher instability risk. Adjustments were limited, as ASA class, BMI, cognitive status, neurological disease, surgeon volume, soft-tissue repair, component orientation, and spinopelvic parameters were unavailable. The three cohorts also differed in demographic, surgical, and implant-related features, and only some of these variables could be included in the statistical models. Event numbers were limited, especially in the modular dual-mobility group. The shorter follow-up and lower number at risk in the modular dual-mobility group should be considered when interpreting 10-year estimates for this construct, as shown by the patients at risk detailed in the figures. Revision for dislocation reflects surgically treated instability and may not capture closed reductions not requiring component exchange. In addition, standard cups with 32 mm heads were not included. This was intentional, because the aim was to compare dual mobility with a contemporary large-head standard cup strategy rather than with small-head conventional THA. However, our findings should not be interpreted as guidance for small acetabula in which a ≥36 mm head is not technically feasible, where dual mobility may remain a relevant stability-enhancing option. Finally, restriction to cementless, non-metal-on-metal THA in regional residents improves internal consistency but may limit generalizability.

These limitations are balanced by a large regional registry cohort, long-term follow-up, minimized loss to revision surveillance, and a contemporary comparison restricted to cementless THA for FNF. Unlike most previous studies, monoblock and modular dual-mobility constructs were analyzed separately and compared with standard cups using femoral heads ≥ 36 mm, while aseptic failure, revision for dislocation/instability, and death-adjusted cumulative incidence were evaluated.

## 5. Conclusions

In this regional registry study of cementless THA for FNF, monoblock and modular dual-mobility cups did not provide superior aseptic failure-free survival compared with standard cups using femoral heads ≥ 36 mm, up to 10 years. Revision for dislocation/instability was rare and comparable across constructs. When a large-head standard cup is technically feasible, routine use of dual mobility should therefore be based on patient-specific instability risk rather than presumed construct superiority. Randomized clinical trials would be ideal to confirm these findings, also including small acetabula, clinical dislocations treated without revision, and implant-specific long-term failure mechanisms of modular versus monoblock dual-mobility designs.

## Figures and Tables

**Table 1:** Demographic, implant-related, and surgical features of the three study cohorts.

Features	Monoblock Dual-Mobility Cups *n* = 1220	Modular Dual-Mobility Constructs *n* = 408	Standard Cups with Femoral Heads ≥ 36 mm *n* = 3199	*p*-Value
Age, years				<0.001
Mean (SD)	72.6 (9.5)	75.4 (11.3)	69.0 (9.7)	
Min, max	32.0, 99.0	32.0, 99.0	21.0, 97.0	
Median (Q1, Q3)	74.0 (67.0, 79.0)	77.0 (70.0, 84.0)	71.0 (64.0, 76.0)	
Age class, years				<0.001
<40	4 (0.3%)	3 (0.7%)	24 (0.8%)	
40–49	21 (1.7%)	8 (2.0%)	107 (3.3%)	
50–59	95 (7.8%)	36 (8.8%)	397 (12.4%)	
60–69	245 (20.1%)	54 (13.2%)	927 (29.0%)	
70–79	589 (48.3%)	146 (35.8%)	1398 (43.7%)	
≥80	266 (21.8%)	161 (39.5%)	346 (10.8%)	
Sex				<0.001
Female	799 (65.5%)	259 (63.5%)	1555 (48.6%)	
Male	421 (34.5%)	149 (36.5%)	1644 (51.4%)	
BMI class				0.113
Underweight	21 (2.9%)	10 (3.1%)	47 (1.9%)	
Normal weight	355 (49.1%)	153 (46.9%)	1117 (45.2%)	
Overweight	269 (37.2%)	118 (36.2%)	1009 (40.9%)	
Obese	78 (10.8%)	45 (13.8%)	296 (12.0%)	
Unknown	497	82	730	
Femoral head material				<0.001
Fourth generation composite ceramic	326 (26.8%)	178 (43.8%)	2794 (87.7%)	
Cobalt-Chrome	308 (25.3%)	217 (53.4%)	128 (4.0%)	
Stainless steel	369 (30.3%)	9 (2.2%)	1 (0.0%)	
Third-generation alumina	181 (14.9%)	2 (0.5%)	152 (4.8%)	
Oxidized Zirconium	32 (2.6%)	0 (0.0%)	111 (3.5%)	
Unknown	4	2	13	
Stem neck modularity				<0.001
Fixed neck	1109 (91.0%)	374 (91.7%)	2526 (79.0%)	
Modular neck	110 (9.0%)	34 (8.3%)	673 (21.0%)	
Unknown	1	0	0	
Surgical approach				<0.001
Posterolateral	943 (77.7%)	295 (72.8%)	931 (29.4%)	
Lateral	211 (17.4%)	73 (18.0%)	1712 (54.0%)	
Anterior	59 (4.9%)	35 (8.6%)	498 (15.7%)	
Other	0 (0.0%)	2 (0.5%)	29 (0.9%)	
Unknown	7	3	29	

Values are reported as *n* (%) unless otherwise indicated. Percentages were calculated excluding unknown values. BMI, body mass index; Q1, first quartile; Q3, third quartile; SD, standard deviation.

**Table 2:** Reasons for aseptic failure within 10 years according to acetabular construct: percentages in brackets.

Cause of Aseptic Failure	Monoblock Dual-Mobility Cups *n* = 1220	Modular Dual-Mobility Constructs *n* = 408	Standard Cups with Femoral Heads ≥ 36 mm *n* = 3199
Periprosthetic fracture	11 (33.3)	6 (54.5)	25 (25.5)
Dislocation/primary instability	5 (15.2)	3 (27.3)	21 (21.4)
Stem aseptic loosening	2 (6.1)	1 (9.1)	20 (20.4)
Unknown	3 (9.1)	1 (9.1)	10 (10.2)
Cup aseptic loosening	8 (24.2)	0 (0.0)	2 (2.0)
Implant breakage	0 (0.0)	0 (0.0)	6 (6.1)
Heterotopic ossification	1 (3.0)	0 (0.0)	4 (4.1)
Total aseptic loosening	1 (3.0)	0 (0.0)	4 (4.1)
Unknown, outside region	0 (0.0)	0 (0.0)	4 (4.1)
Pain without loosening	1 (3.0)	0 (0.0)	1 (1.0)
Acetabular fracture	1 (3.0)	0 (0.0)	0 (0.0)
Other	0 (0.0)	0 (0.0)	1 (1.0)
**Total aseptic failures**	**33 (2.7)**	**11 (2.7)**	**98 (3.1)**

Values are reported as *n* (%). Percentages refer to aseptic failures within each construct group; in the final row, percentages refer to the total number of implanted THAs within each construct group. THA, total hip arthroplasty.

## Data Availability

The data presented in this study are available on reasonable request from the corresponding author.

## Abbreviations

The following abbreviations are used in this manuscript:

ASA	American Society of Anesthesiologists
BMI	Body Mass Index
CI	Confidence Interval
DM	Dual Mobility
FDA	Food and Drugs Administration
FNF	Femoral Neck Fracture
HA	Hydroxyapatite
HR	Hazard Ratio
IQR	Interquartile Range
ISAR	International Society of Arthroplasty Registries
RIPO	Registro dell’Impiantologia Protesica Ortopedica
RR	Risk Ratio
SD	Standard Deviation
THA	Total Hip Arthroplasty
